# Supplementary material for: A Bibliometric Analysis on Research Progress of Earthworms in Soil Ecosystems
Source: Biology (Basel). 2024 May 28;13(6):385. doi: 10.3390/biology13060385 (PMC11201220; doi:10.3390/biology13060385)
Supplement: Supplementary file 1 [file biology-13-00385-s001.zip › biology-2993919-supplementary.pdf]

Supplementary material

Table S1. Top 10 institutions with bursts in terms of publication records related earthworm researches from 1980-2022

Institutions	Strength	Begin	End	1980-2022
Univ Chinese Acad Sci (China)	34.53	2017	2022	
Chinese Acad Sci (China)	28.10	2017	2022	
Tech Univ Darmstadt (Germany)	24.10	1999	2010	
CSIRO (Australia)	19.66	1992	1999	
Shandong Agr Univ (China)	18.79	2018	2022	
Ohio State Univ (USA)	18.67	1998	2008	
INRA (France)	17.38	2013	2016	
Vrije Univ Amsterdam (Netherlands)	16.54	2003	2016	
Sorbonne Univ (France)	16.42	2019	2022	
Nanjing Agr Univ (China)	15.71	2018	2022	

Table S2. Authors with burst in terms of publication records related earthworm researches in the last five years

Authors	Strength	Begin	End	1980-2022
WANG J	25.00	2018	2022	
WANG JH	16.62	2018	2022	
ZHU LS	13.29	2018	2022	
HU F	9.60	2018	2022	
LE BAYON RC	6.26	2018	2019	
DU ZK	5.86	2018	2020	
ZHU YG	5.90	2019	2022	
ZHU D	5.90	2019	2022	
DOMINGUEZ J	4.85	2019	2022	
BRIONES MJI	4.85	2019	2020	
SHAO MA	8.19	2020	2022	
YANG Y	6.10	2020	2022	
LI M	6.10	2020	2022	
BARETTA D	5.84	2020	2022	

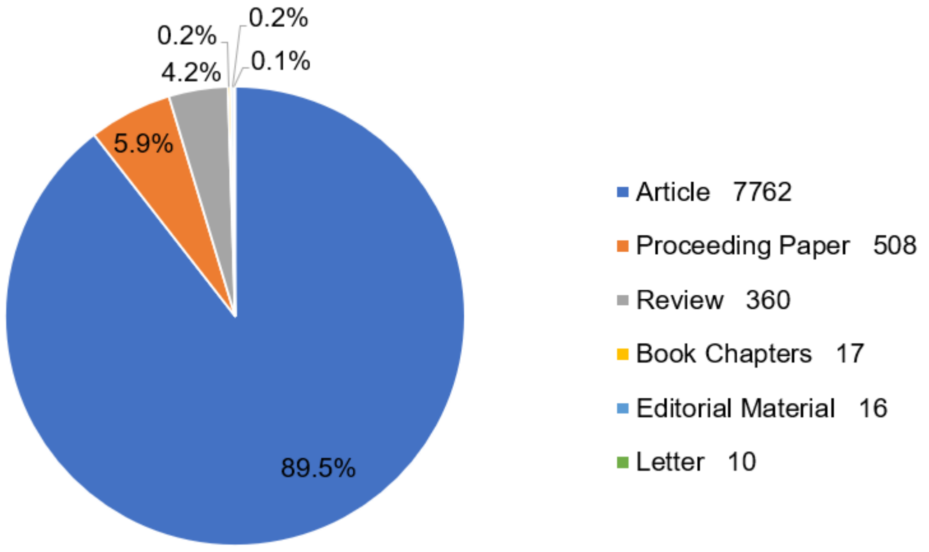

Figure S1. Literature types of earthworm related publication collected in WoS from 1900 to 2022

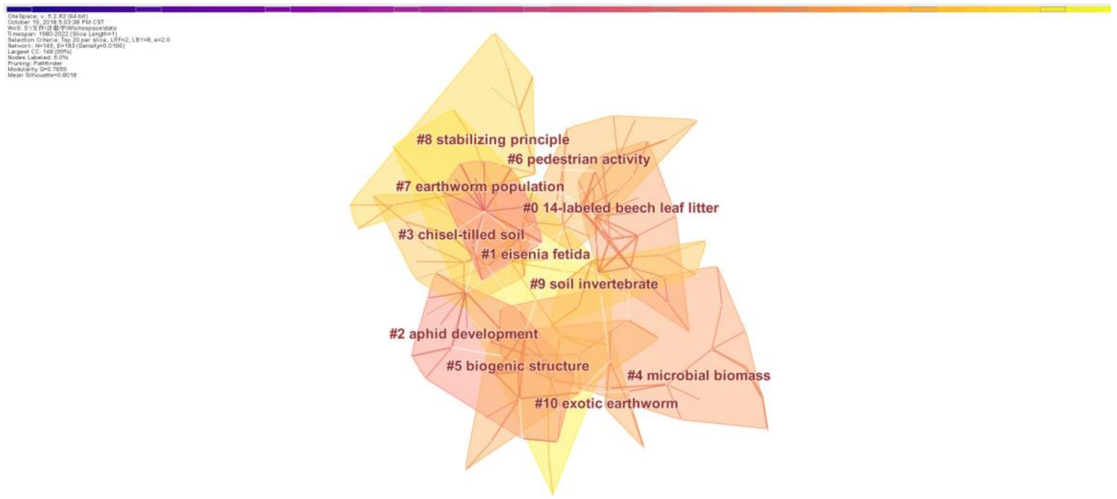

Figure S2. The co-occurrence network of terms in earthworm related papers collected in WoS
